# Nitrogen Balance and Protein Quality Ingestion in Pregnant Women: Characterizing a Nutritional Scenario in a Pilot Study in Mexico

**DOI:** 10.1002/fsn3.71757

**Published:** 2026-05-29

**Authors:** Adriana Granich‐Armenta, Alejandra Cantoral, Rosa María Mariscal‐Moreno, Gina Castiblanco‐Rubio, Ivonne Ramírez‐Silva, Melanie Y. Mendoza Jimenez, E. Angeles Martinez‐Mier, Laura Ávila‐Jiménez, Juan A. Rivera Dommarco

**Affiliations:** ^1^ Health Department Universidad Iberoamericana Mexico City Mexico; ^2^ Department of Dental Public Health and Dental Informatics Indiana University School of Dentistry Indianapolis Indiana USA; ^3^ Center for Health and Nutrition Research National Institute of Public Health Cuernavaca Morelos Mexico; ^4^ Undersecretariat of Health Policies and Population Welfare National Public Health Service in Morelos Cuernavaca Morelos Mexico; ^5^ Social Security Mexican Institute Regional Hospital #1 in Cuernavaca Cuernavaca Morelos Mexico; ^6^ Center for Population Health Research National Institute of Public Health Cuernavaca Morelos Mexico

**Keywords:** diet quality, duplicate portion method, nitrogen balance, pregnancy, protein intake

## Abstract

Nitrogen balance (NB) reflects protein metabolism and varies with diet quality. We aimed to estimate the NB using objective standardized methods and evaluate the adequacy and quality of protein intake in Mexican pregnant women. This pilot study was nested in the MAS‐Lactancia cohort and included 13 pregnant women in their third trimester. Diet was assessed through the duplicate portion method (DPM) and food diaries on two non‐consecutive days. A registered dietitian analyzed photos, recipes, and portion sizes to assess dietary quality using NOVA classification. DPM samples were analyzed by Kjeldahl method to determine nitrogen content. On the same days, 24 h‐urine was collected to measure urea and total nitrogen excretion. NB was calculated per day. The Kruskal Wallis test compares the characteristics of the participants according to NB. Mean age (SD) was 27.9 (4.2) years, at 33 (3.4) gestational weeks. Median nitrogen intake was 6.75 g/day (IQR: 6.40–9.74), representing 0.64 g protein/Kg body weight by day (IQR: 0.45–0.92). Median NB was 1.32 g (IQR: −0.15 to 2.63), with 30.7% of women in negative, 23.2% in neutral, and 46.1% in positive balance (+2). Diet quality showed high carbohydrate intake (> 70% of total calories), mainly from ultra‐processed foods. Protein came in moderate amounts from foods containing 15%–95% of the edible portion. Negative NB was more common among women with higher BMI and lower education. Over half of pregnant women were in negative or neutral NB, indicating insufficient protein intake from limited sources and low diet quality.

## Graphical abstract

We assessed dietary intake and protein adequacy among pregnant women using the duplicate portion method and urinary nitrogen excretion. Nitrogen balance was used to evaluate protein status, showing that poorer diet quality and higher BMI were associated with negative nitrogen balance, while better diet quality was linked to positive balance.

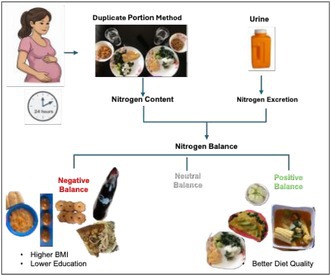

**Abbreviations**
AACC: American Association of Cereal Chemistry
BMI: body mass index
DPM: duplicate portion method
EN: excreted nitrogen
NB: nitrogen balance
RDA: recommended dietary allowances
SES: socioeconomic status
UUN: urinary urea nitrogen

## 1 Introduction

Nitrogen balance (NB) is a measure of lost or retained nitrogen and an indicator of protein metabolism and nutritional status (Bartholomae and Johnston 2023). NB is a reliable and objective tool for assessing protein intake necessary for an individual, as dietary protein contains approximately 16% nitrogen by weight, with 1 g of nitrogen representing an approximate intake of 6.25 g of protein (Goldstein 1998). Depending on specific nutritional goals, NB is expected to be neutral or positive (Pupim et al. 2013).

NB is estimated from the difference between the amount of nitrogen consumed and the amount of nitrogen excreted per day (Weiler et al. 2023). Nitrogen excretion can be objectively estimated through 24 h urine collections (Corder et al. 2024). This measurement also provides information about protein intake. A positive balance indicates that more nitrogen is retained, which occurs during growth, pregnancy, or recovery from injury. On the other hand, negative NB happens when more nitrogen is lost and occurs during malnutrition, illness, or injury (Pupim et al. 2013). Negative NB is associated with dietary patterns characterized by low protein intake, which fail to meet daily recommendations, and inadequate intake of essential amino acids are linked to low birth weight and other complications (Pérez‐Tepayo et al. 2020).

Non‐pregnant adults are usually in neutral balance (±0.5 g/day accounting for unmeasured nitrogen losses from sweat, skin desquamation, hair and nail growth, and respiration). During neutral NB, whole body protein synthesis is equal to whole body protein degradation (Madhumathi et al. 2000). During pregnancy, women undergo significant physiological changes to support fetal growth and development; therefore, a positive NB is expected, reflecting a net anabolic state (Tijerina Sáenz et al. 2014). A negative nitrogen balance during pregnancy can compromise both maternal and fetal health, leading to maternal malnutrition and intrauterine growth restriction (Allman et al. 2019). It reflects a net catabolic state resulting from inadequate protein intake or excessive protein losses (Pupim et al. 2013). NB depends on the quality of the diet, not only on its protein content and composition, but also on energy and adequate intake of micronutrients (Calvez et al. 2024).

Dietary questionnaires do not commonly assess dietary quality directly, so indexes or scales must be calculated to estimate this information. The NOVA scale classifies foods according to their degree of processing, showing that greater consumption of ultra‐processed foods is associated with higher energy intake, lower micronutrient content, lower protein consumption, and overall reduced diet quality (Martini et al. 2021; Monteiro et al. 2018). Higher ultra‐processed food consumption is associated with substantially lower quality diet among children and adults (Liu et al. 2022); this is particularly important as these foods are increasingly consumed by women of reproductive age (Granich‐Armenta et al. 2024; de Oliveira et al. 2022).

In Mexico, the general population's intake of protein has been reported as adequate or high; these reports mainly use dietary questionnaires to evaluate adequacy (Pérez‐Tepayo et al. 2020). According to the National Health and Nutrition Survey, the protein intake in the general population increased from 12% to 16% of total caloric intake from 2012 to 2016 (Aburto et al. 2022), this data did not show the quality of the protein and the main sources. In the case of pregnancy, the Nutrition Survey of 1988 reported that 14.7% of the calories came from proteins, representing an average intake of 65.04 g/day (Flores et al. 1998). A cross‐sectional study of pregnant women from the northwestern metropolitan area of Mexico City reported a median protein intake of 70 g/day, corresponding to 113% of the Recommended Dietary Allowance (RDA). However, the authors noted that the main dietary protein sources were of lower quality, such as cereals (Rosa Isela et al. 2005). Some other cross‐sectional analyses using dietary questionnaires have reported intakes in pregnancy around 70–80 g/day, suggesting an adequate intake; however, there is still limited evidence assessing the quality of protein intake, particularly among pregnant women (Reyes‐López et al. 2021; Romero‐Villanueva et al. 2022).

To date, no objective and accurate estimation of protein intake during pregnancy has been conducted using gold‐standard methods. We aimed to (1) assess the NB during pregnancy using the duplicate portion method (DPM) and urea nitrogen from 24‐h urine collections, and (2) evaluate the adequacy and quality of protein intake in a sample of Mexican pregnant women.

## 2 Materials and Methods

### 2.1 Design and Setting

This pilot study was nested within an ongoing birth cohort study (MAS‐Lactancia) that began in 2016 and recruited pregnant women until 2024. More information about the cohort's protocol is available in a former publication (Ramirez‐Silva et al. 2021). All pregnant women provided written informed consent to become study participants within the cohort. The protocol was approved by the INSP Ethics in Research Committee and Research Commission (approval 1281), and the IMSS National Committee of Scientific Research (#R2015‐785‐107).

### 2.2 Participants and Sample

From October to December 2023, women were contacted if they met the cohort eligibility criteria (age 18–39 years, more than 16 weeks of gestation, residence in Cuernavaca and surrounding areas, and planned delivery at IMSS Clinic No. 1) and fulfilled the additional eligibility requirements of the Validation of the 24‐h dietary recall using the duplicate diet method and biomarkers (such as fluoride, sodium, and potassium) in urine (24‐h and spot samples) subproject, which included suitability for participation in the duplicate‐diet validation protocol and urinary biomarker assessment. This required the absence of medical conditions such as preeclampsia, gestational diabetes, or exposures that could interfere with fluoride metabolism or urinary biomarker validity, such as multiple pregnancy, active smoking, or chronic second‐hand smoke exposure, certain chronic diseases, use of specific medications like fluoxetine or antihypertensive agents, or occupational exposure to industrial pollutants.

This subgroup was selected to ensure high‐quality exposure assessment and complete biomarker data, which constituted the main analytical framework of the sub‐study. No formal statistical power calculation was conducted, as this work was designed as an exploratory pilot study based the sample on previous studies on dietary data and nitrogen balance during pregnancy (Martinez‐Mier et al. 2009; Mojtahedi et al. 2002). The final sample size of 13 pregnant women was additionally constrained by logistical and funding considerations, including the resources available to implement the duplicate‐diet protocol and provide food purchase vouchers to participants.

### 2.3 Dietary Assessment

Dietary assessments were done in two non‐consecutive weekdays through a DPM (Kim et al. 1984; Trijsburg et al. 2015) and a food diary with photographic aids (Rebro et al. 1998). DPM involves collecting a second equal portion of all the food or drink consumed within 24 h and laboratory analyses of the food's composition to estimate the nutrient content with higher accuracy (Trijsburg et al. 2015). Dietary data were collected on weekdays only; however, this approach may not fully capture usual dietary intake, as dietary patterns often differ between weekdays and weekends. For this pilot study, participants received food vouchers to purchase the additional foods needed for DPM. Each participant was provided with labeled containers to collect duplicated food intake portions (one for solid foods, one for water, and one for beverages, per day of collection). Additionally, the participants recorded the food intake through a diary and photos of each food consumed. They were also asked to record in a standardized form all the food and beverages consumed, portion sizes, recipes for each meal, meal type (breakfast, lunch, dinner, and snacks) for both homemade and restaurant meals. Before eating the meal, participants were asked to take a photo with their mobile phones, and to include information of the meal's date and time, before and after each meal's consumption (Table S2). All these procedures were supported by trained dietitians who assisted pregnant women at home from 7 am to 5 pm. The trained dietitians weighed all homemade meals.

Food quality was estimated using the participant‐provided photos. Trained personnel compared images taken before and after consumption. After the dietary data were gathered from participant‐provided photos, foods and food products were categorized according to the NOVA classification system. NOVA classifies foods based on the degree and purpose of processing in four groups. Group 1 includes unprocessed or minimally processed foods, which are natural items modified only through methods like drying or cooking. Group 2 consists of processed culinary ingredients derived from foods or natural sources, such as salt and sugar. Group 3 includes processed foods that have had substances like sugar or salt added to Group 1 foods. Lastly, Group 4 includes ultra‐processed products, which are industrial formulations containing multiple ingredients, including additives, combined with processed substances (Monteiro et al. 2018). Additionally, since our trained dietitians weighed and recorded all foods consumed, a description of the main sources of protein, including type of food source, edible portion and the amount consumed, was captured and used to estimate the protein intake using the nutrient composition tables named Mexican Food Database (BAM, for its acronym in Spanish) (Ramírez Silva et al. 2021).

### 2.4 Food Sample Analysis

The 24 h collection food samples by the DPM were analyzed to determine the macronutrient content of proteins, crude fiber, and fats. Carbohydrate content was calculated by subtracting the sum of the other components from 100% (Mæhre et al. 2018). To obtain homogeneous samples, all foods collected each day for each participant were blended using a food processor (Hamilton Beach 70740, 8‐Cup Food Processor; Hamilton Beach Brands, Glen Allen, VA, USA). A volume of deionized water was added until a pureed consistency was obtained. Samples were frozen at −80°C ± 2°C until their analysis. Liquid samples were analyzed without pretreatment, and the added moisture was corrected for in the calculations.

Proximate analysis of samples was conducted following the approved methods of the American Association of Cereal Chemistry (AACC), determined by measuring the lost weight after 3 h in an oven at 105°C. Protein was obtained by Kjeldahl method. To obtain protein content, the nitrogen obtained was multiplied by factor conversion of 6.25. Crude fat was determined by the Soxhlet method (American Association of Cereal Chemistry 2025). Ash measurement was made by gravimetric method using a muffle maintained at 550°C for 3 h. The assessment of crude fiber was done using acid digestion followed by a loss on ignition using a fiber analyzer. Moisture, ash, protein, fat, and fiber content were measured according to specific AACC methods: 44–15.02, 08–01.01, 46–16.01, 30–25.01, and 32–10.01, respectively (American Association of Cereal Chemistry 2025). The nutritional value of the duplicate plate was calculated using conversion factors according to EU Regulation No 1169/2011: (1) Carbohydrates (except polyols): 4 kcal·g ^−1^; (2) Protein, 4 kcal·g ^−1^; (3) Fat, 9 kcal·g ^−1^; (4) Fiber, 2 kcal·g^−1^ (Europeo et al. 2011).

To estimate diet nitrogen through the Kjeldahl method, the grams of protein consumed from both solid foods and beverages were divided by the conversion factor of 6.25 (Mariotti et al. 2008). Additionally, protein intake per kilogram of body weight was determined using the participant's third trimester weight measured at enrollment for this pilot study (Elango and Ball 2016).

### 2.5 Nitrogen Excretion in 24 h‐Urine

Participating pregnant women watched a video that outlined the procedure for collecting 24 h‐urine samples to collect total urinary volume. Participants were given round wide mouth plastic containers with 1 gal capacity to store urine. They were instructed to discard the first urine of the day and then collect all urine over the course of the day and night (24 h) until the next morning's first urine. To determine the levels of Urinary Urea Nitrogen (UUN), the samples were analyzed using the Spectrophotometry method at a certified clinical laboratory (Watt and Chrisp 1954). The UUN (mg/dL) was then multiplied by the total urine volume (dL) to obtain g of UUN in 24 h. To estimate the excreted nitrogen (EN), the following formula was applied:

$$\mathrm{EN}\;\left(g\right)=\left(\mathrm{UUN}\;\text{in}\;g/L\times 0.46\times \text{urine volume}\;L\right)+4$$

where the factor 0.46 represents the proportion of nitrogen in urea, and the additional 4 g account for estimated nitrogen losses from other non‐urea sources (William Mitch 1989).

### 2.6 Nitrogen Balance

After calculating nitrogen values from the analysis of DPM meals, beverages, and 24‐h EN, we estimated NB as dietary nitrogen intake minus nitrogen excretion. The NB was categorized as negative if NB was lower than zero, neutral if NB was between 0 and < 2, and positive if the NB was ≥ 2 (Duggleby and Jackson 2002).

### 2.7 Covariates

Sociodemographic characteristics such as age, education, socioeconomic status (SES), occupation, and marital status were obtained from the cohort's database. Education was assessed as years of completed formal schooling. SES was classified using the cohort's standardized household asset‐based index and categorized as low, medium, or medium–high (AMAI 2024). Occupation was categorized as formal employment (paid work with contract and social security) or housewife/informal work/student. Household characteristics (housing acquisition, household size, number of children < 6 years, bedrooms, and overcrowding) were obtained from the cohort questionnaire based on the AMAI (2024). Anthropometric measurements such as weight (kg) were measured by study‐trained personnel, following Lohman's Anthropometry Manual as a reference (Lohman et al. 1988). Maternal weight was recorded using an electronic scale (Tanita, model 1582, Illinois, USA), which was accurate to the nearest 100 g.

### 2.8 Statistical Analysis

Continuous variables were reported according to their distribution, means and standard deviations (SD) for normally distributed variables. Medians and interquartile ranges (IQR) were used for variables with skewed distribution. Results for nitrogen intake and excretion and NB were presented as medians with interquartile ranges (IQR) and for urinary nitrogen as means with standard deviations (SD). Categorical variables were presented as frequencies and proportions. Kruskal Wallis test was used to compare the characteristics of the participants according to NB (negative, neutral, and positive). The paired Wilcoxon signed‐rank test estimated the difference in the DPM from both days analyzed by each participant, and a paired *t*‐test was used to compare urine variables between days. Comparisons in baseline variables between the 13 women of this analytical sample and the rest of the cohort were done. All analyses were done using Stata 14.0 (StataCorp LP, College Station, TX, USA) (StataCorp 2015).

## 3 Results

The 13 participants in this study had a mean (SD) gestational age of 33 (3.4) weeks, a mean age of 27.9 (4.2) years, and a mean educational level of 12.7 (4.6) years. The majority reported medium SES (58.3%). Half of the participants had formal work (53.8%), and 84.6% were married or cohabitated with a partner. No statistical differences in sociodemographic variables among the cohort and the analytical sample were observed (Table S1).

### 3.1 Urinary Results

The collection of 2 days of the 24 h‐urine for the 13 participants is presented in Table 1. The mean (SD) volume of the 24 h‐urine collection was 1301.15 (528.05) mL/day, and the mean UUN estimated in the laboratory was 468.50 (137.04) mg/dL (±137.04), which reflects a 24‐h mean UUN of 5.30 (2.08) g/24 h (±2.08). Mean EN was 6.63 (±0.93) g/day. No statistical differences were found within participants in the 2 days of urine collection.

**TABLE 1:** Urine analysis results from the 24‐h urine collection, *n* = 13.

	Day 1	Day 2	Average	*p* *
	Mean (SD)	Mean (SD)	Mean (SD)	
Total urinary volume (mL/24 h)	1256.92 ± 621.65	1336.25 ± 523.12	1301.15 ± 528.05	0.44
Urinary urea nitrogen (mg/dL)	486.38 ± 165.32	430.66 ± 131.75	468.50 ± 137.04	0.40
24‐h urinary urea nitrogen (g/24 h)	5.08 ± 2.25	5.16 ± 2.03	5.30 ± 2.08	0.42
Excreted nitrogen (g)	6.53 ± 1.01	6.57 ± 0.93	6.63 ± 0.93	0.43

Abbreviation: SD, standard deviation.

* *p*‐value obtained using the paired *t*‐test to assess statistically significant differences between Day 1 and Day 2 (*p* < 0.05).

### 3.2 Chemical Composition of Diet

The results of the DPM chemical composition are presented in Table 2. The table reports the median (IQR) estimations by day and by type of food (solid, beverages, and total food stuffs collected). The analysis composition showed a median nitrogen of 6.75 g/day (IQR: 6.40–9.74), which reflects a median protein of 44.21 g/day (40.05–60.89), and by body weight of 0.64 g/kg/day (IQR: 0.45–0.92), as mean (SD) third trimester body weight was 72.4 kg (±11.49).

**TABLE 2:** Chemical composition of foods and beverages from the DPM during third trimester of pregnancy (*n* = 13).

	Day 1	Day 2	Average	*p* *
	Median (IQR: 25th—75th percentile)			
*Solid Food*				
Nitrogen (g)	6.98 (5.61–8.69)	7.08 (4.59–8.90)	6.62 (6.01–9.01)	0.73
Protein (g)	43.66 (35.07–54.35)	44.26 (28.74–55.67)	41.37 (37.59–56.36)	0.73
Estimated consumed protein by body weight (g/kg/day)	0.67 (0.51–0.86)	0.63 (0.37–0.93)	0.64 (0.45–0.92)	0.72
Carbohydrates (g)	155.20 (137.54–180.17)	156.47 (128.49–198.85)	157.51 (147.24–183.68)	0.77
Fats (g)	9.56 (4.21–11.17)	8.93 (4.33–18.91)	8.54 (6.87–13.03)	0.85
Fiber (g)	23.17 (12.6–24.94)	19.71 (14.78–23.79)	19.56 (15.53–23.87)	0.88
*Beverages*				
Nitrogen (g)	0.25 (0–0.74)	0 (0–0.69)	0.38 (0–0.99)	0.34
Protein (g)	1.6 (0–4.62)	0 (0–4.32)	1.92 (0–6.23)	0.35
Estimated consumed protein by body weight (g/kg/day)	0.02 (0–0.06)	0 (0–0.06)	0.02 (0–0.06)	0.60
Carbohydrates (g)	37.77 (20.07–46.06)	40.73 (30.72–62.15)	41.16 (30.35–48.89)	0.27
Fats (g)	0 (0–0.63)	0 (0–0)	0.19 (0–0.32)	0.22
Fiber (g)	0.0	0.0	0.0	0.61
*Total* (*solids and beverages*)				
Nitrogen (g)	7.32 (6.22–9.10)	7.08 (5.21–9.57)	6.75 (6.40–9.74)	0.68
Total protein (g)	45.78 (38.92–56.89)	44.26 (32.57–59.83)	42.21 (40.05–60.89)	0.68
Estimated consumed protein by body weight (g/kg/day)	0.67 (0.51–0.86)	0.63 (0.37–0.92)	0.64 (0.45–0.92)	0.72
Total energy from protein (kcal)	183.12 (155.68–227.56)	177.04 (130.30–239.34)	168.84 (160.22–243.58)	0.62
Protein contribution to total estimated consumed energy (%)	20.06 (15.68–20.90)	15.34 (13.30–18.38)	18.18 (15.39–19.93)	0.38
Total carbohydrate (g)	201.02 (183.60–218.51)	205.00 (171.00–240.53)	210.32 (186.75–226.67)	0.55
Total energy from carbohydrate (kcal)	804.08 (734.40–874.04)	820.00 (684.00–962.12)	841.30 (747.02–906.7)	0.55
Carbohydrate contribution to total estimated consumed energy (%)	71.21 (69.69–75.83)	71.91 (69.24–81.41)	74.65 (72.39–75.49)	0.81
Total fats intake (g)	10.21 (4.59–11.19)	8.93 (4.33–19.45)	8.61 (7.9–13.03)	0.83
Total energy from fats (kcal)	91.89 (41.31–100.71)	80.37 (39.01–175.05)	77.53 (71.1–117.31)	0.83
Fats contribution to total estimated consumed energy (%)	8.14 (5.41–11.02)	7.84 (3.50–13.45)	7.00 (5.30–11.71)	0.65
Total estimated consumed energy (kcal)	1108.90 (916.69–1184.55)	1171.29 (882.98–1295.99)	1162.23 (992.83–1248.50)	0.08
Fiber total (g)	23.17 (12.6–25.56)	19.71 (14.78–24.01)	20.88 (15.53–23.87)	0.87

Abbreviation: IQR, interquartile range (25th–75th percentile).

* *p*‐value obtained using the Wilcoxon paired signed‐rank test to assess statistically significant differences between Day 1 and Day 2 (*p* < 0.05).

For the rest of the nutrients, the median for carbohydrates, fats, and fiber was 210.32 g/day (IQR: 186.75–226.67), 8.61 g/day (IQR: 7.9–13.03), 20.88 g/day (IQR: 15.53–23.87), respectively. The median total estimated consumed energy was 1162.23 kcal/day (IQR: 992.83–1248.50), with more than 70% of the calories coming from carbohydrates, and less than 7% from fats. Beverage analysis shows that they mainly contain carbohydrates and less than 0.5 g of nitrogen. No statistical differences in diet composition for both days were noted.

### 3.3 Nitrogen Balance

With the information from the Urinary results and the Chemical Composition of Diet Table 3 presents the NB analysis. Median NB in the pregnant women of this pilot study was 1.32 g (IQR: −0.15, 2.63), however a third of them were in a negative NB and only 46% achieve a positive NB of +2 as recommended (Joint WHO/FAO/UNU Expert Consultation 2007); the remaining participants were in neutral balance.

**TABLE 3:** Nitrogen balance during third trimester of pregnancy (*n* = 13).

	Day 1	Day 2	Average	*p* *
	Median (IQR: 25th—75th percentile)			
Nitrogen intake estimated by DPM (g)	7.32 (6.22, 9.10)	6.60 (5.02, 8.76)	6.75 (6.40, 9.74)	0.68
Excreted nitrogen in 24‐h urine (g)	6.59 (5.80, 6.88)	6.80 (6.34, 7.12)	6.88 (6.38, 6.98)	0.43
Nitrogen balance (g)	1.41 (−0.08, 2.68)	0.76 (−1.39, 1.99)	1.32 (−0.15, 2.63)	0.57
Women with negative balance (%)	30.7	41.6	30.70	
Women with > 2 g of nitrogen balance (%)	38.4	25.0	46.15	

Abbreviation: IQR, interquartile range (25th–75th percentile).

* *p*‐value obtained using the Wilcoxon signed‐rank test to assess statistically significant differences between Day 1 and Day 2 (*p* < 0.05).

### 3.4 Participant's Characteristics Related to NB

Table 4 presents the comparison of participants' main sociodemographic, anthropometric, and dietary characteristics according to the categorization of NB. We observed a tendency in age and years of education, where women in negative NB were older and had fewer years of schooling compared to those in the neutral and positive balance (though not statistically significant). For those in the neutral NB, the number of cohabitants and children younger than 6 years was higher compared to the other two groups (also non statistically significant). Women in the negative NB presented a higher BMI and weight (33.9 kg/m^2^ SD ±5.8, 82.02 kg ±SD 13.5, respectively), and a lower gestational age (31 ± 2.7 weeks), compared to those in a positive NB.

**TABLE 4:** Characteristics of the participants related to NB.

	Analytic sample	Negative NB	Neutral NB	Positive NB	*p*
	*n* = 13	*n* = 4 (30.7%)	*n* = 3 (23%)	*n* = 6 (46.1%)	
Age (years)^a^	27.9 (4.2)	29.0 (6.0)	27.3 (0.57)	27.5 (4.37)	0.82
Education (years)^a^	12.7 (4.6)	9.5 (5.19)	14.6 (4.9)	14.0 (3.5)	0.26
*Socioeconomic status* ^b^					
Low	2 (16.7)	1 (33.3)	0 (0)	1 (16.6)	0.99
Medium	7 (58.3)	1 (33.3)	2 (66.6)	4 (66.6)	
Medium–High	3 (25)	1 (33.3)	1 (33.3)	1 (16.6)	
*Occupation*					
Housewife—Informal‐student	6 (46.2)	2 (50)	2 (66.7)	2 (33.3)	0.79
Formal	7 (53.8)	2 (50)	1 (33.3)	4 (66.7)	
*Marital status* ^b^					
Single	2 (15.4)	1 (25)	0 (0)	1 (16.6)	0.99
Married—Cohabitated	11 (84.6)	3 (75)	3 (100)	5 (83.7)	
House characteristics					
*House acquisition*					
With no pay	3 (23)	0 (0)	1 (33.3)	2 (33.3)	0.81
Rented	7 (54)	3 (75)	1 (33.3)	3 (50)	
Own house	3 (23)	1 (25)	1 (33.3)	1 (16.6)	
Num persons living in the house	5.1 (4.1)	6 (5.4)	8.6 (4.1)	2.8 (1.3)	0.11
Num children < 6 years in the house	0.38 (0.65)	0.5 (0.57)	0.66 (1.15)	0.16 (0.40)	0.56
Num bedrooms in the house	2 (1.15)	1.7 (0.95)	3.3 (1.5)	1.5 (0.54)	0.11
*Overcrowding hose*					
Yes	2 (15.4)	1 (25)	1 (33.3)	0 (0)	0.26
No	11 (84.7)	3 (75)	2 (66.6)	6 (100)	
*Anthropometrics*					
Weight (kg)	72.4 (11.5)	**82.02 (13.5)**	**74.4 (12.2)**	**64.9 (2.08)**	**0.02**
Height (cm)	157.1 (5.13)	155.5 (3.2)	157.6 (2.3)	157.8 (7.1)	0.78
BMI	29.4 (5.07)	**33.9 (5.8)**	**29.9 (4.6)**	**26.1 (1.6)**	**0.08**
Gestational age (weeks)	33 (3.4)	**31 (2.7)**	**31 (4)**	**35 (1.9)**	**0.06**
*Daily dietary protein*					
Grams	48.75 (15.32)	**35.31 (6.84)**	**37.85 (5.0)**	**63.17 (7.77)**	**0.01**
Grams per body weight	0.70 (0.27)	**0.44 (0.11)**	**0.52 (0.10)**	**0.97 (0.11)**	**< 0.01**

*Note:* Overcrowding hose is defined as the # persons living in the house/# bedrooms. Statistical significance is indicated using bold values (*p* < 0.1).

^a^ Means and standard deviations for continuous variables, *p*‐value for Wilcoxon test, bolds indicated *p* values < 0.1.

^b^ Percentages for categorical variables, *p*‐value for exact Fisher test, bolds indicated *p* values < 0.1.

Finally, it is important to highlight that the mean (SD) protein intake derived from the chemical composition analysis was 35.31 g/day (SD ±6.84) representing 0.44 g/body weight/day (SD ±0.11), 37.85 g/day (SD ±5.0) representing 0.52 g/body weight/day (SD ±0.10), and 63.17 g/day (SD ±7.77) representing 0.97 g/body weight/day (SD ±0.11) for negative, neutral and positive NB, respectively. For the first two (negative and neutral) this intake is almost half of the recommendation (Table 4).

### 3.5 Photo‐Based Food Diary, Quality of the Diet

Table 5 shows the frequency of food consumed by NOVA group according to the food photo diary. The food intake was distributed as follows: 30.8%, 0.96%, 25%, and 40.3% corresponded to NOVA 1 “Unprocessed or minimally processed foods”, NOVA 2 “culinary ingredients”, NOVA 3 “processed foods”, and to NOVA 4 “ultra‐processed foods”, respectively. We also listed the most consumed foods or beverages by the pregnant women by NOVA group. As seen, the main sources of protein with high amino acid content were chicken, egg, milk, fresh cheese, and plain yogurt. However, the edible part of the chicken consumed by participants is low as they mainly buy chicken with bones, such as wings, which implies that the average portion consumed in a meal is only around 43 g (providing an average of 7 g of protein). In the case of eggs, participants commonly consume two whole fried eggs mainly at breakfast. For milk, they reported consuming whole milk in a cup of 250 mL, usually adding chocolate or sugar. Cheese was consumed as an additive, like spread cheese on an average of 30 g per serving. In the case of yogurt, participants used to buy 1 L presentation and serve it in a small cup of 120 mL. The daily diet evaluation shows that the previously mentioned animal protein sources are not all consumed the same day (Table S2). Even though the participants have sources of protein of high amino acid content, as meats and dairy representing 49.3% of total proteins, according to the nutrient composition tables, the portions bought, or the amount consumed (considering the edible part), is not sufficient. Additionally, legumes which are considered a good vegetarian source of proteins, only represent 4.2% of the diet protein contribution (Table 3), in contrast to the Mexican Dietary Guidelines that recommend consuming 1–1½ cups of legumes daily for pregnant women, corresponding to approximately 10%–15% of total daily energy intake based on a 2200 kcal diet (SSA, INSP, GISAMAC, U 2023). Finally, the brands of sausages and ham consumed by participants, commonly considered protein sources, were low‐cost options with limited nutritional quality; some reports indicate that these brands include flours or starches in their formulation instead of meat (Procuraduría Federal del Consumidor 2025).

**TABLE 5:** Most consumed food by pregnant women using NOVA classification.

Dummy‐remove						
NOVA 1	Contribution (%)	30.80%				
	Most frequent foods (%)	**Chicken parts (edible portion): wing (42.5%), feet (42.5%), drumstick (42.5%), pelvic bones (15%), breast ribs (8%). Average intake of 43 g per serving lunch**.	**Egg (edible portion): whole egg (95%). Average intake of 43 g per serving breakfast**.	**Whole milk. Average intake of 250 mL per serving breakfast or dinner**.	Raw apple	Raw cucumber
		15.4	13.5	11.5	7.7	7.7
	Participant's meal sample where you can find the listed food	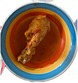 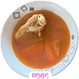	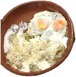	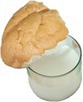	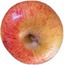	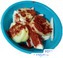 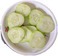
NOVA 2^a^	Contribution (%)	0.84%				
NOVA 3	Contribution (%)	25%				
	Most frequent foods (%)	Corn tortilla	Mexican Roll—Bolillo	Regular sour cream	**Fresh cheese. Average intake of 30 g per serving breakfast of lunch**.	**Plain whole milk yogurt. Average intake of 120 mL per serving breakfast**.
		25.5	18.6	11.6	11.6	9.3
	Participant's meal sample where you can find the listed food	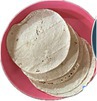	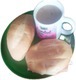	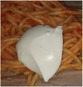	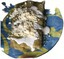	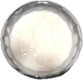
NOVA 4	Contribution (%)	40.30%				
	Most frequent foods (%)	Cookies	White bread	Brewed black iced tea pre‐sweetened with sugar	Pre‐packed Turkey ham‐Deli, Luncheon meat	Jelly dessert
		17.4	17.4	13	13	8.7
	Participant's meal sample where you can find the listed food	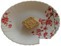 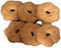 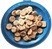	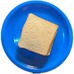	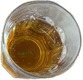	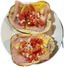 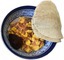	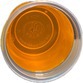

*Note:* Bold indicates food with high amino acids value in proteins.

^a^ Contribution NOVA 2 (it is not listed in top foods).

To compare the methods used in this pilot study, we present in Table 6 the recommendations on protein intake in the last trimester of pregnancy and the estimations done by using the nutrient composition tables and the chemical analysis of the DPM. As we can observe, the traditional dietary methods that use composition tables yield results closer to the recommendations; the intake estimated by the tables is below more than 10% of the recommendations and is probably overestimating the protein intake when comparing with the DPM, considered as the gold standard.

**TABLE 6:** Comparison of methods in estimating protein intake.

	Daily grams of protein		Daily grams of protein per body weight	
	Median	IQR	Median	IQR
Recommendation^a^	75.35	70.95, 79.31	1.1	
24 H recall	66.1	49.2, 75.6	0.88	0.68, 1.12
DPM	42.21	(40.05–60.89)	0.64	(0.45–0.92)

^a^ Institute of medicine.

## 4 Discussion

To our knowledge, this is the first study in Mexican pregnant women that assessed NB using quantitative and objective methods, including DPM, which is considered the gold standard in nutritional epidemiology (Nakamura et al. 2004; Ning et al. 2022), and with the collection of 24 h‐urine to estimate the main output of nitrogen. Our results showed that during the third trimester, a third of the women were in negative NB and only 40% achieved a positive NB of +2 needed to cover fetal demands (Duggleby and Jackson 2002). During the third trimester of pregnancy, when fetal demand is at its highest, a positive nitrogen retention can reach up to 1.3 g/day of retained nitrogen (King 2000). Therefore, NB is expected to increase toward the end of pregnancy due to a more efficient utilization of dietary proteins (Mojtahedi et al. 2002). Negative or neutral NB during the third trimester may compromise fetal growth, as it suggests the body is breaking down more protein than it is consuming, potentially reflecting insufficient dietary protein intake or heightened physiological demands (Herring et al. 2018; Mojtahedi et al. 2002).

Our DPM analysis shows that participants were consuming half of the recommended protein intake for pregnancy. Our estimated median (IQR) intake of 0.64 (0.45–0.92) g/kg/day: 0.45, 0.92 is almost half of the daily recommendation of 1 g/kg/day during singleton pregnancy (Institute of Medicine (US) 2005). Our results contrast with what has being reported previously by dietary questionnaires in the Mexican population, where protein intake was above 60–80 g/day and reporting a good adequacy (Flores et al. 1998; Reyes‐López et al. 2021; Romero‐Villanueva et al. 2022; Rosa Isela et al. 2005). However, this analysis is consistent with other reports across diverse worldwide contexts, showing that many women fail to meet dietary recommendations during pregnancy. For example, a Brazilian report shows that pregnant women did not modify their food choices compared to non‐pregnant women, resulting in persistently inadequate nutrient intake: 97% had inadequate iron intake, 78% had inadequate folate intake, and 59% had inadequate vitamin B6 intake (Dos Santos et al. 2014). In rural Bangladesh, worsening household food insecurity was linked to reduced maternal dietary diversity and lower consumption of nutrient‐rich foods, particularly animal‐source foods, fruits, and vegetables (Na et al. 2016). Then, in Uganda's Karamoja sub‐region, more than half of pregnant women had inadequate energy intake and the majority failed to meet recommendations for protein, calcium, iron, zinc, and folate, with diets dominated by starchy staples and low in animal‐source foods (Muggaga et al. 2023).

Our chemical analysis gave a detailed view of the macronutrient distribution of the foods and beverages from DPM. The protein intake appeared somewhat below the recommended level for pregnant women, which is around 71 g per day according to the Institute of Medicine (IOM) guidelines (Institute of Medicine (US) 2005). Sufficient protein intake is essential in pregnancy to support fetal growth, maternal tissue expansion, and increased blood volume. On the other hand, carbohydrate intake on both days was around the recommended 175 g per day for pregnant women (Institute of Medicine (US) 2005), but it represents more than 70% of total calories, which would be not the ideal distribution.

Fat intake was lower than expected, providing less than 10% of the calories. Dietary fat is important during pregnancy for the development of the fetal brain and eyes (mainly by polyunsaturated fatty acids), as well as for the absorption of fat‐soluble vitamins (Schwartz et al. 2025). Ensuring a balance of healthy fats could improve maternal and fetal outcomes.

As noted previously, the women in this analysis did not meet the recommended protein intake. Moreover, comparison of the chemical analysis of DPM samples with estimates derived from nutrient composition tables and dietary records showed that the latter methods tend to overestimate protein intake. It is acknowledged that questionnaires such as the Food Frequency Questionary and 24H recalls may be overestimating the intake of protein during pregnancy (Steinemann et al. 2017). Additionally, we observed that the overestimation of protein intake may be related to the edible portion used in the calculations. The literature has recognized this is a crucial problem when estimating the intake, particularly when the portion consumed do ton exclude parts as bones or peels (Asna et al. 2024). Also, if the estimations use is done with an average edible portion, this could imply an overestimation of the meat consumed. As an example, chicken bought and consumed by pregnant women in this pilot study, as wings, feet, drumstick, pelvic bones, breast ribs, contain bones and the edible portions are between 8% and 42.5% (Abdullah et al. 2017; Chambers et al. 2017; Qiaoxian et al. 2020; Santana et al. 2020; Acabonac Farms n.d.).

Another explanation of the overestimation when using the nutrient composition tables is that this source of information requires a constant update of the new products on the market. Substitute foods, especially processed or lower‐quality options, like certain cheeses or sausages, often have nutrient profiles that differ substantially from the foods they replace. This can lead to inaccuracies if tables use generic or average values that do not reflect the specific substitute consumed. Many food composition tables aggregate data from multiple brands or use median values for food categories, which may not capture the full range of nutrient content found in substitute or lower‐quality products. This can result in under‐ or overestimation of nutrients such as sodium, saturated fat, or protein (Chen et al. 2024). As an example, the brand of ham reported by the women of this study has a protein content that does not compare to the traditional ham made from pork meat; the ones reported indicate in the label that the main ingredients are pork, fat, and starch. So even though ham is commonly perceived as a significant source of protein, this selection may lead to poor dietary choices, especially for people in vulnerable situations. Moreover, these processed meats are characterized by high levels of saturated fats, sodium, and additives such as nitrites, which are associated with health risks (Sirini et al. 2022).

The NOVA classification done with the photo diary showed that minimally processed foods (NOVA 1) contributed to one‐third of total caloric intake, meanwhile a significant portion of their diet (40.3%) consists of NOVA 4 (ultra‐processed foods) which may contribute to the higher intake of added sugars, saturated fats, and sodium; and except for ham, the foods reported in this category are mainly starchy foods, which may explain why the amount of carbohydrate consume is very high in this population.

The macronutrient content of beverages can offer additional insight into overall diet quality, particularly if they are made with whole milk as a source of protein and calcium; however, the analysis revealed that most beverages primarily contributed to simple carbohydrates, such as soda.

This study has some limitations. First, the sample size may limit the generalizability of the findings to other populations. Second, the assessment of NB was conducted only during the third trimester, and thus, may not reflect protein intake and utilization throughout the entire pregnancy. Also, the study relied on urinary nitrogen excretion as a primary measure of nitrogen loss, which may not capture all routes of nitrogen loss such as fecal and miscellaneous nitrogen losses that were not measured directly, potentially underestimating total nitrogen excretion (Maroni et al. 1985). Third, protein metabolism was assessed using nitrogen balance rather than direct measures of protein turnover, which require specialized indicators such as stable isotope tracer techniques and may provide a more precise characterization of the increased protein synthesis and degradation known to occur during pregnancy (Thompson and Halliday 1992). Fourth, dietary intake was assessed on weekdays only, which may not fully reflect usual dietary patterns, as food consumption often differs on weekends due to changes in routines, social activities, and eating behaviors. The exclusion of weekend days may therefore limit the ability to comprehensively capture habitual dietary intake (Tooze et al. 2010). Fifth, the dietary intake and nitrogen balance were assessed only during the third trimester of pregnancy; future research should evaluate these outcomes across different trimesters to allow comparison of dietary and metabolic changes throughout gestation. Additionally, maternal body weight was used as a covariate rather than body mass index, which is consistent with standard approaches for estimating protein and nitrogen requirements expressed per kg of body weight; this decision may limit the ability to account for differences in body composition and adiposity and should be considered when interpreting the findings of this pilot study (Johansson et al. 2001). Lastly, the use of food vouchers may have affected dietary behavior, although efforts were made to minimize this through participant‐selected foods and explicit instructions to maintain usual intake (Miguel‐Berges et al. 2022).

Despite the above limitations, this study has several notable strengths. It is, to our knowledge, the first study to assess NB using objective methods in Mexican pregnant women, providing valuable insights into protein utilization during the third trimester. By contrasting the findings with previous dietary records that relied on questionnaires, the study highlights potential inaccuracies in traditional dietary assessment methods. The use of NB as a direct measure of protein utilization offers a more objective and accurate assessment of protein status compared to dietary records alone. Moreover, the DPM was conducted with the support of trained dietitians who assisted participants in recording, weighing foods, and taking photos; therefore, it is unlikely that food collection for the DPM was incomplete or that intake was underestimated.

Assessing with accuracy protein intake is crucial to make a correct interpretation on diet during pregnancy, as proteins are key macronutrients for fetal growth and development, and the mother's blood volume, tissue growth, placenta nutrient transport, and after delivery, milk production (Imdad and Bhutta 2011; Ota et al. 2015). In addition to the amount of protein, quality food consumption during pregnancy is relevant for supporting optimal maternal and child health (Paula et al. 2023).

In conclusion, this study provides novel insights into protein intake and utilization during the third trimester of pregnancy in this sample of Mexican women, revealing that protein intake may be insufficient during the third trimester, as indicated by a negative or neutral NB. Also, our chemical analysis revealed that diet composition is mainly given by carbohydrates with inadequate amounts of protein and very low amounts of fats. This highlights the need for more accurate and objective methods to assess protein status during pregnancy and underscores the importance of diet quality and the selection of protein‐rich foods, which should be emphasized in dietary guidelines for pregnant women.

## Author Contributions

**Adriana Granich‐Armenta:** conceptualization, investigation, writing – original draft, methodology, formal analysis, project administration, data curation, visualization, writing – review and editing, validation. **Alejandra Cantoral:** conceptualization, investigation, funding acquisition, methodology, validation, visualization, writing – review and editing, formal analysis, project administration, supervision, resources, writing – original draft. **Rosa María Mariscal‐Moreno:** conceptualization, investigation, writing – review and editing, methodology, validation, formal analysis, data curation, supervision, resources. **Gina Castiblanco‐Rubio:** funding acquisition, writing – review and editing, methodology, validation, supervision, investigation. **Ivonne Ramírez‐Silva:** writing – review and editing, methodology, investigation. **Melanie Y. Mendoza Jimenez:** writing – review and editing, project administration, investigation. **E. Angeles Martinez‐Mier:** methodology, resources, writing – review and editing. **Laura Ávila‐Jiménez:** investigation, project administration, resources, supervision. **Juan A. Rivera Dommarco:** conceptualization, methodology, validation, supervision.

## Funding

We would like to express our gratitude to the Indiana University for their institutional support and for providing internal funding. Also, to the funding of Consejo Nacional de Ciencia y Tecnología (CONACYT) (Grant number 233439), Fundación Gonzalo Río Arronte (Grant number S687).

## Conflicts of Interest

The authors declare no conflicts of interest.

## Supporting information


**Table S1:** Sociodemographic characteristics of the participants.
**Table S2:** Examples of food intake in 1 day.

## Data Availability

The data that support the findings of this study are available on request from the corresponding author. The data are not publicly available due to privacy or ethical restrictions.
