# Supplementary material for: Nitrogen Balance and Protein Quality Ingestion in Pregnant Women: Characterizing a Nutritional Scenario in a Pilot Study in Mexico
Source: Food Sci Nutr. 2026 May 29;14(6):e71757. doi: 10.1002/fsn3.71757 (PMC13239914; doi:10.1002/fsn3.71757)
Supplement: Supplementary file 1 — Table S1: Sociodemographic characteristics of the participants. Table S2: Examples of food intake in 1 day. [file FSN3-14-e71757-s001.docx]

| **Supplementary table 1. Sociodemographic Characteristics of the Participants** | | | |
| --- | --- | --- | --- |
|  | **General Cohort** | **Analytic Sample** | **p-value*** |
|  | **n=660** | **n=13** |  |
| **Age (years)^a^** | 26.4 (5.02) | 27.9(4.2) | 0.29 |
| **Education (years)^a^** | 12.9 (3.00) | 12.7 (4.6) | 0.81 |
| **Socioeconomic Status ^b^** |  |  |  |
| Low | 212 (32.1) | 2 (16.7) | 0.24 |
| Medium | 221 (33.5) | 7 (58.3) | 0.06 |
| Medium-High | 227 (34.4) | 3 (25) | 0.48 |
| **Occupation^b^** | | |  |
| Housewife - Informal-Student | 324 (49.3) | 6 (46.2) | 0.82 |
| Formal | 334 (50.7) | 7 (53.8) | 0.82 |
| **Marital Status^b^** |  |  |  |
| Single | 92 (13.9) | 2 (15.4) | 0.88 |
| Married - Cohabitated | 566 (86.1) | 11(84.6) | 0.88 |
| ^a^ Means and standard deviations for continuous variables, p-value for t-test | | | |
| ^b^ Percentages for categorical variables, p-value for exact Fisher test | | | |

| **Supplementary Table 2. Examples of food intake in one day** | | | | |
| --- | --- | --- | --- | --- |
| **Participant´s one day diet** | **Participant A** | **Participant A** | **Participant B** | **Participant B** |
|  | **Meal** | **Duplicate Plate** | **Meal** | **Duplicate Plate** |
| **Breakfast** | 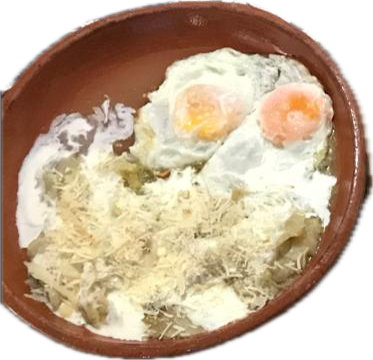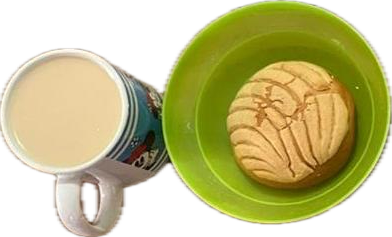 | 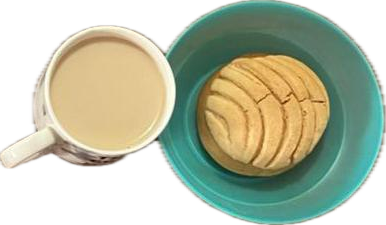 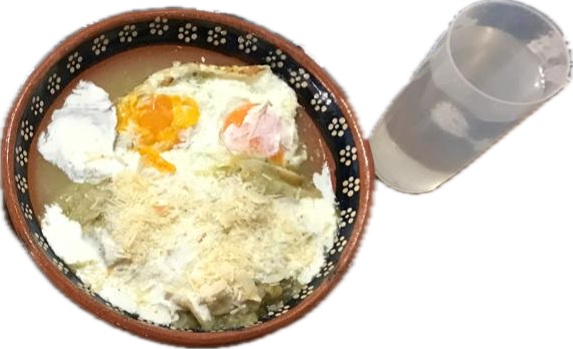 | 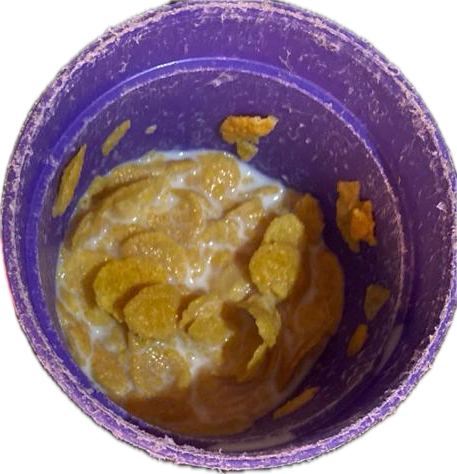 | 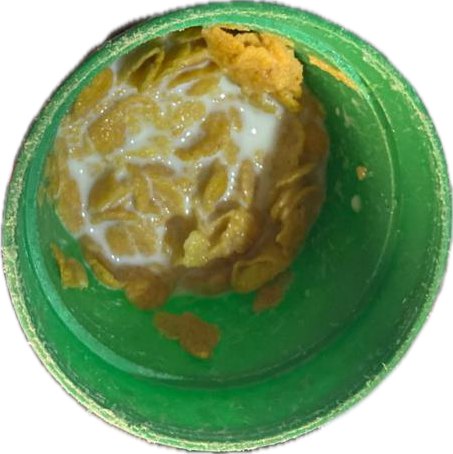 |
| **Morning Snack** | 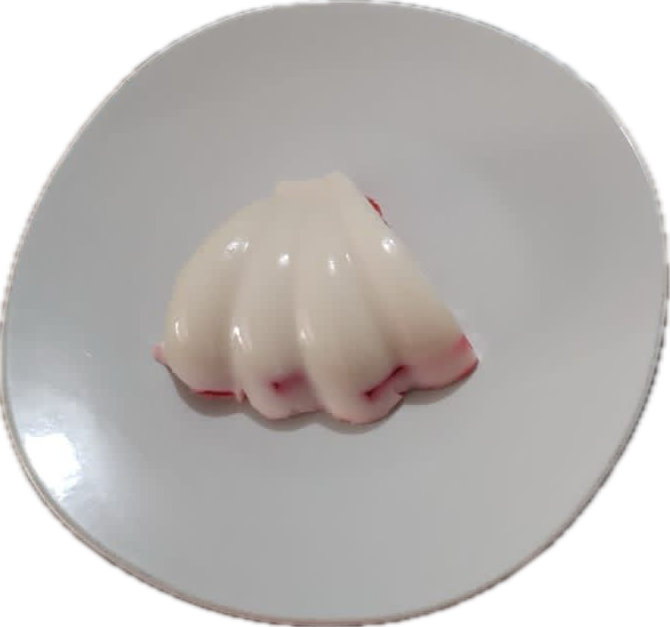 | 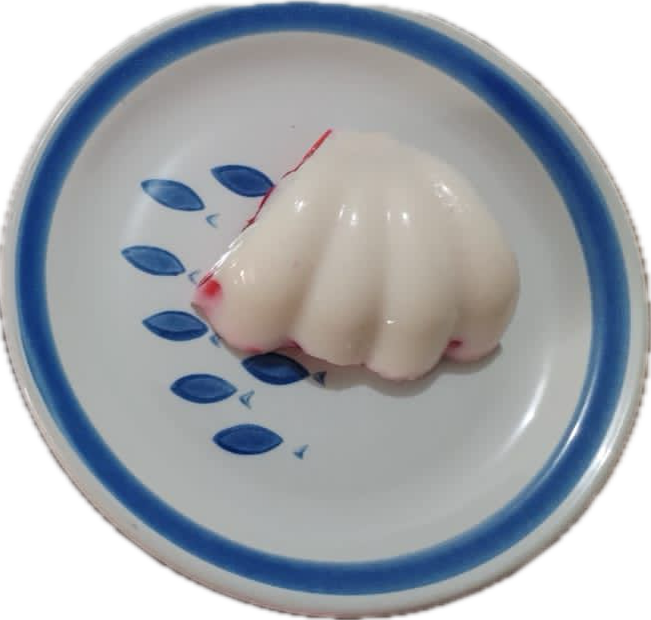 | 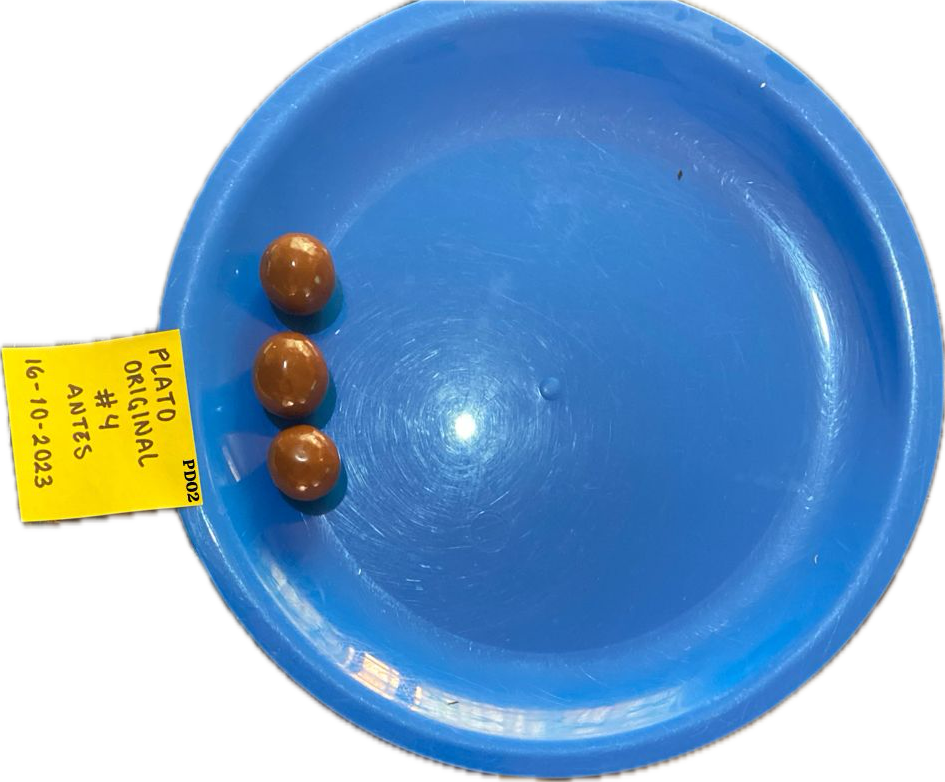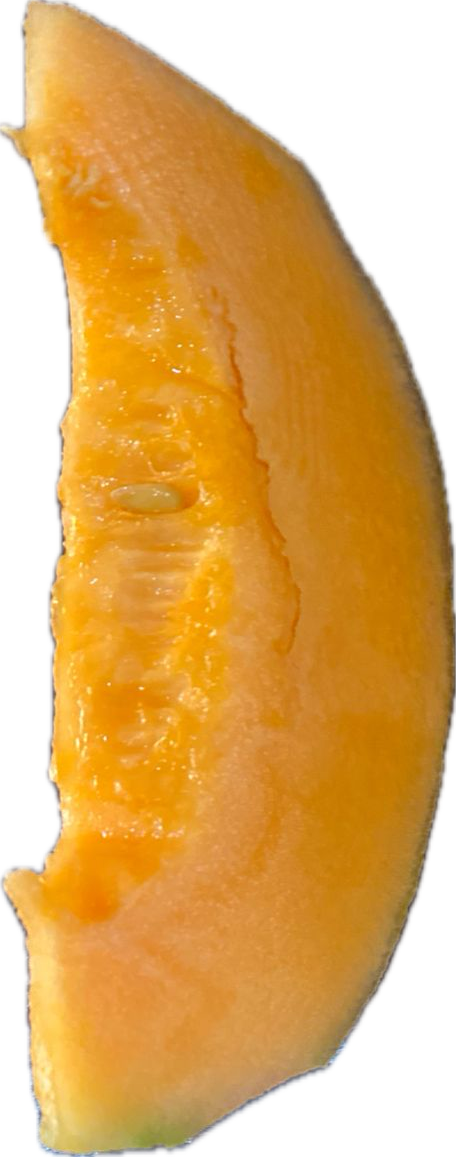 | 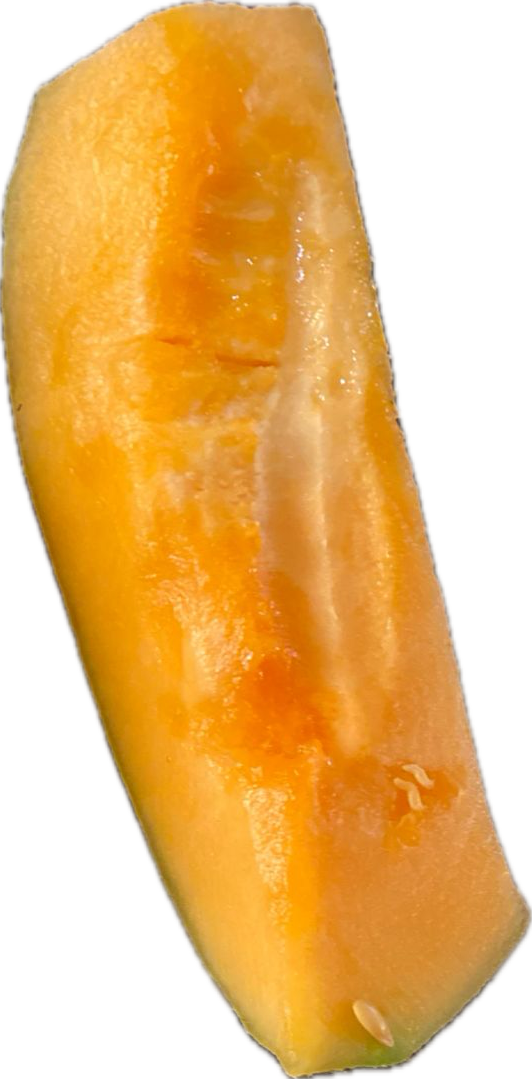 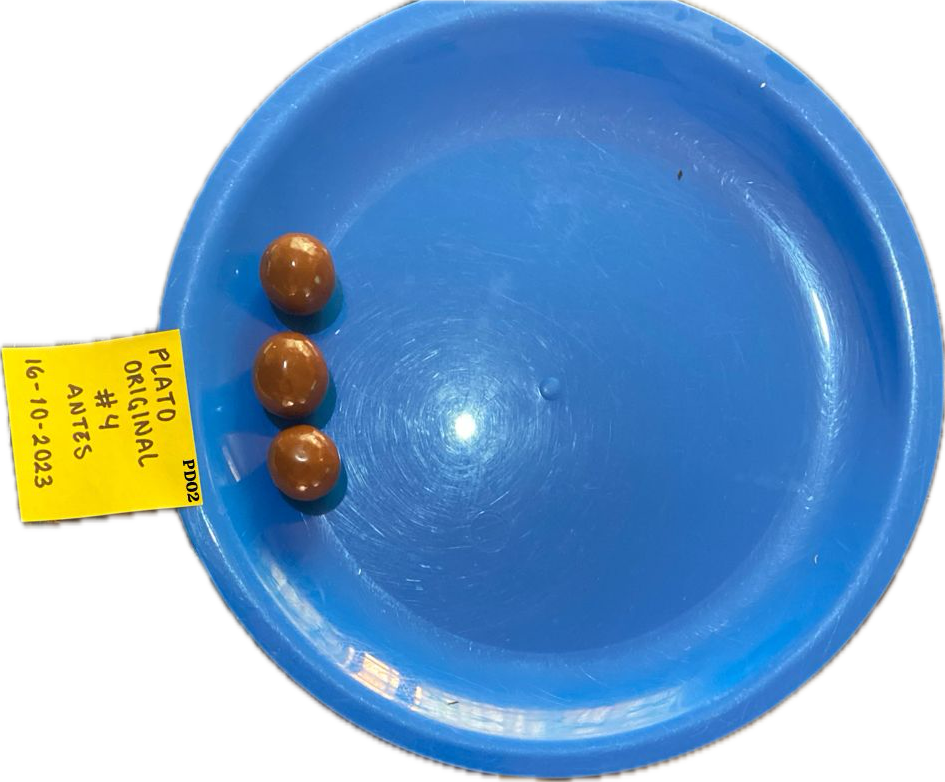 |
| **Lunch** | 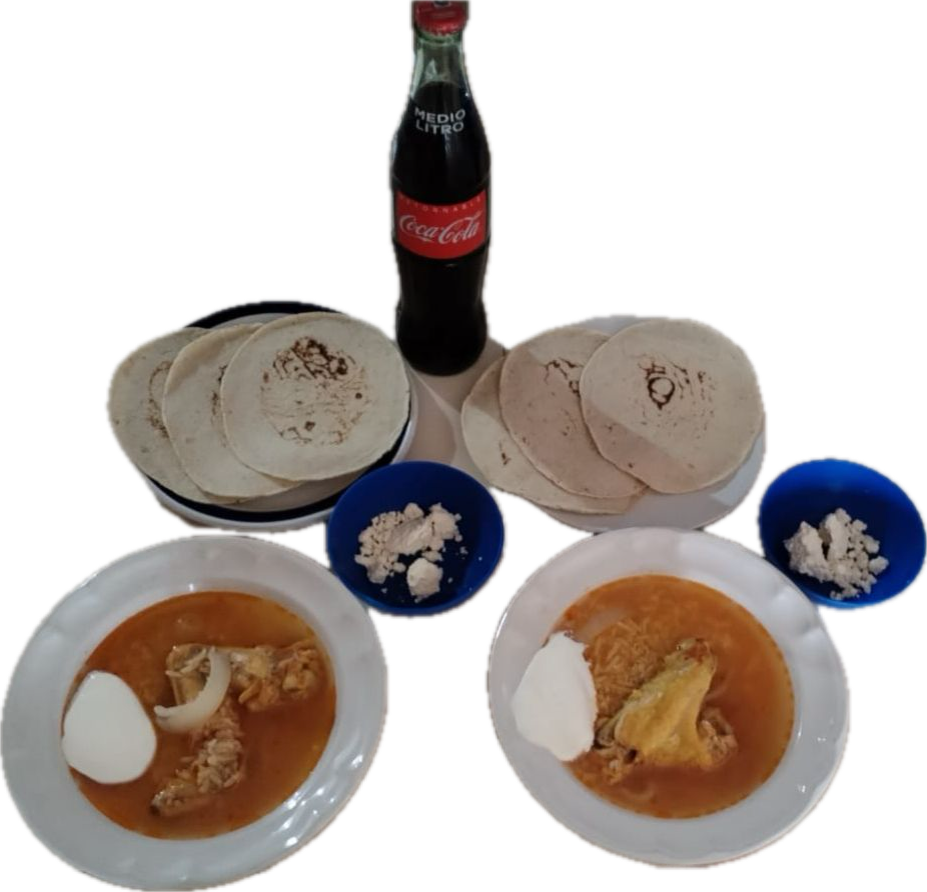 | 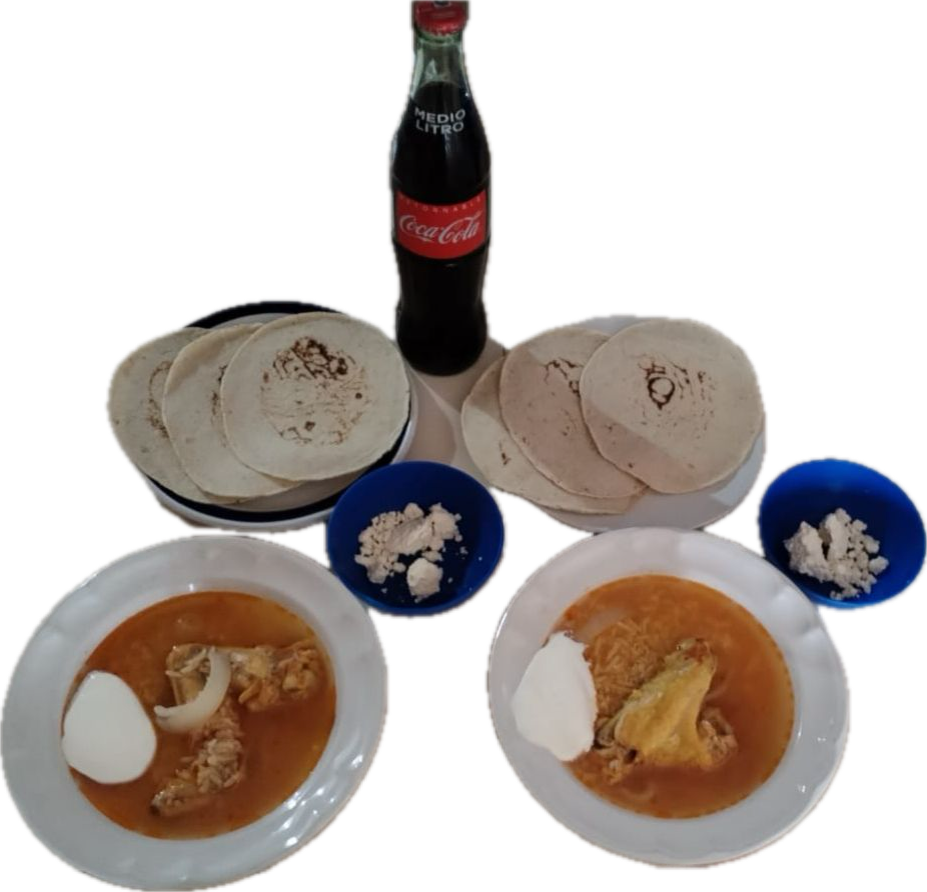 | 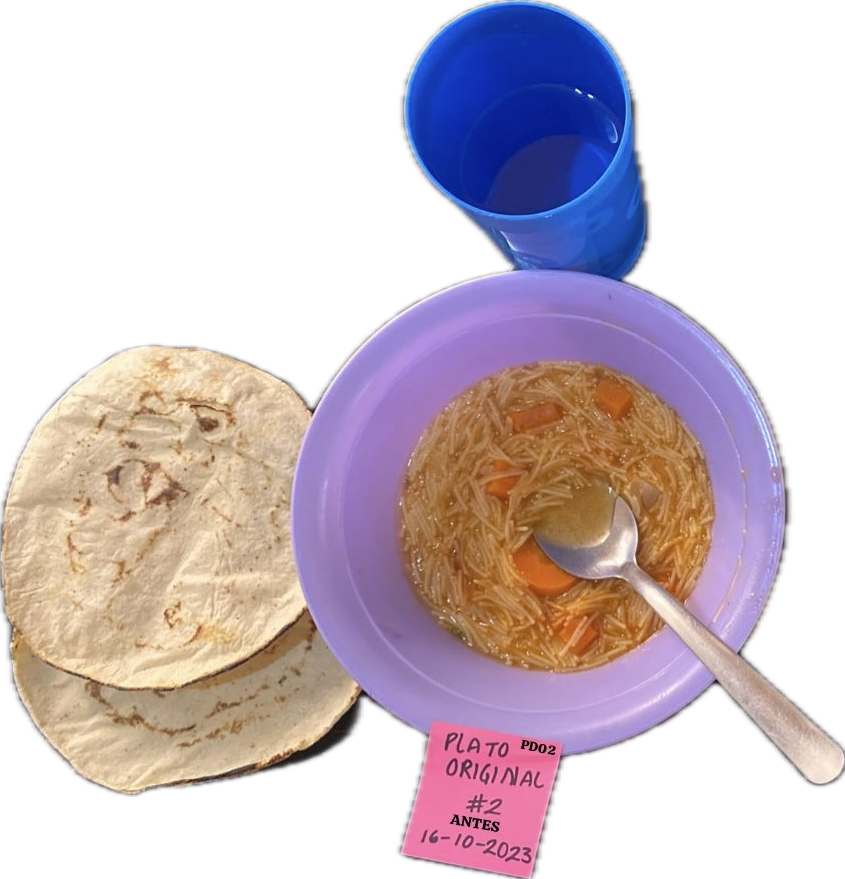 | 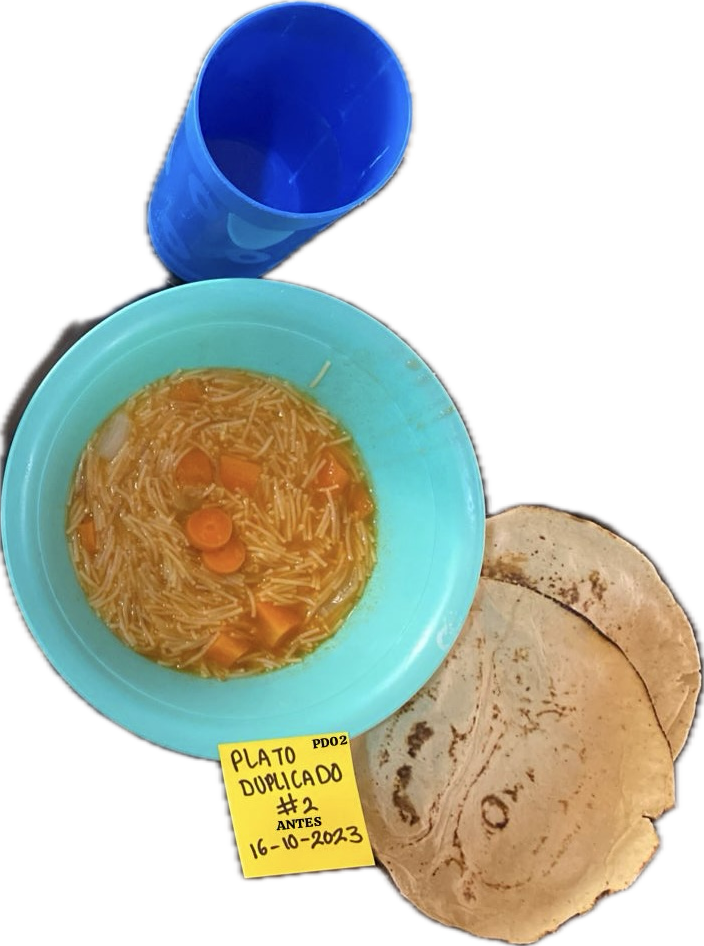 |
| **Afternoon Snack** | **N/A** | **N/A** | 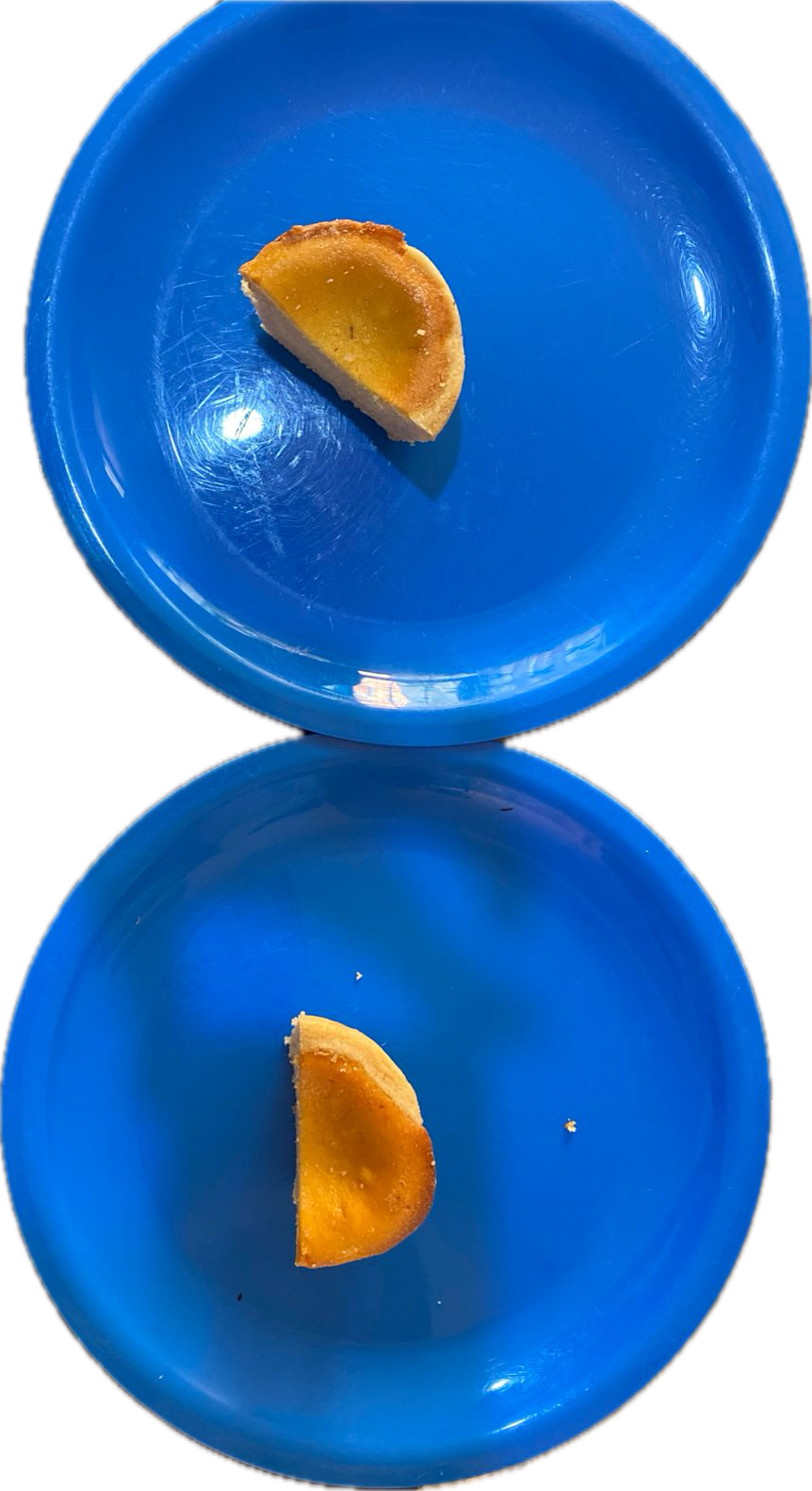 | 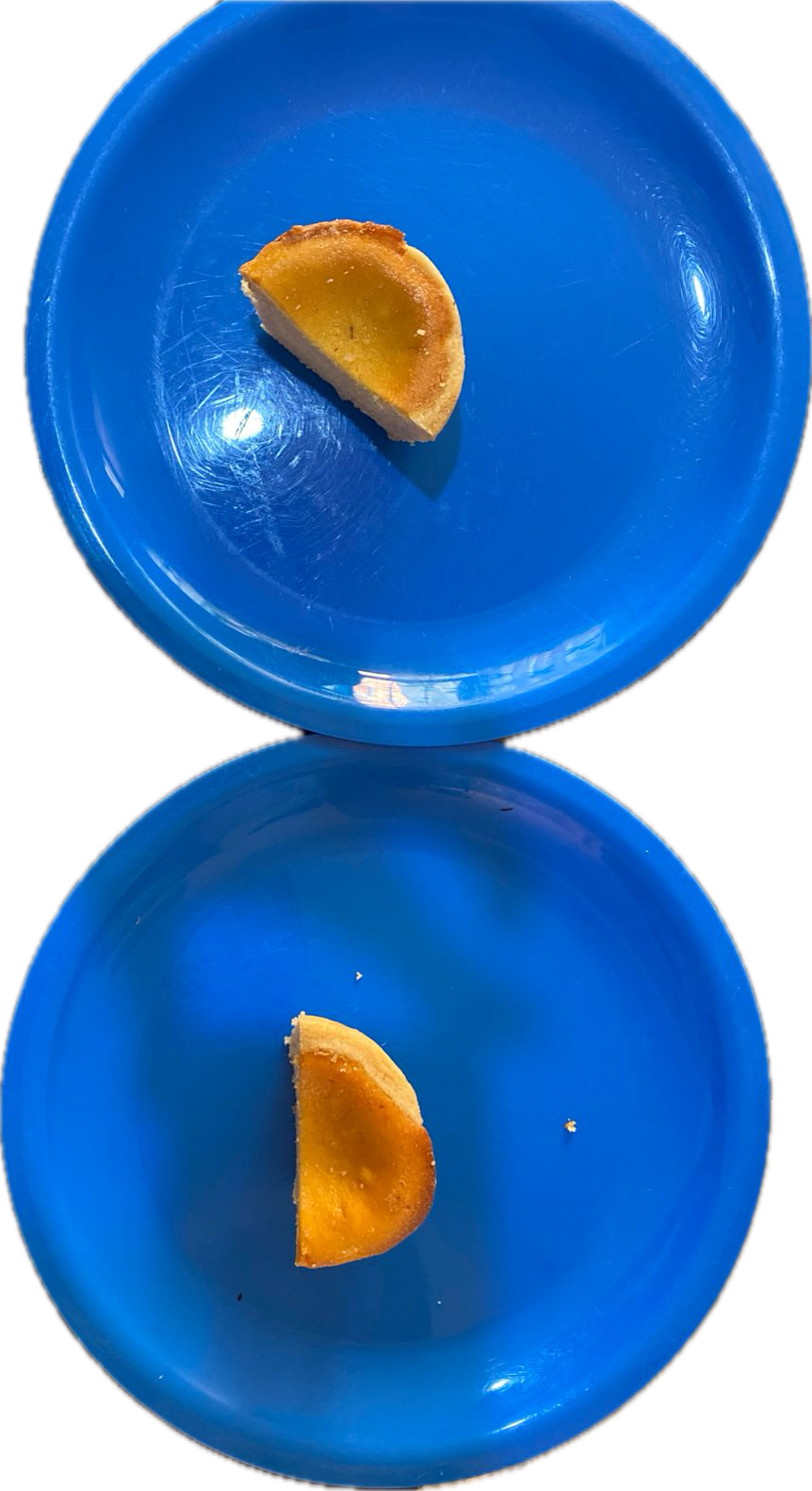 |
| **Dinner** | 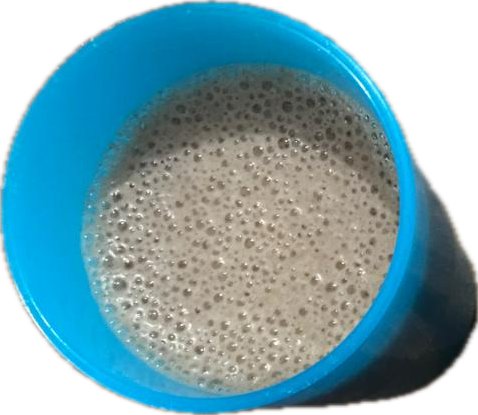 | 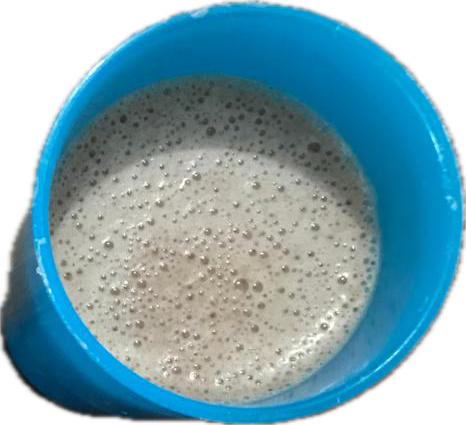 | 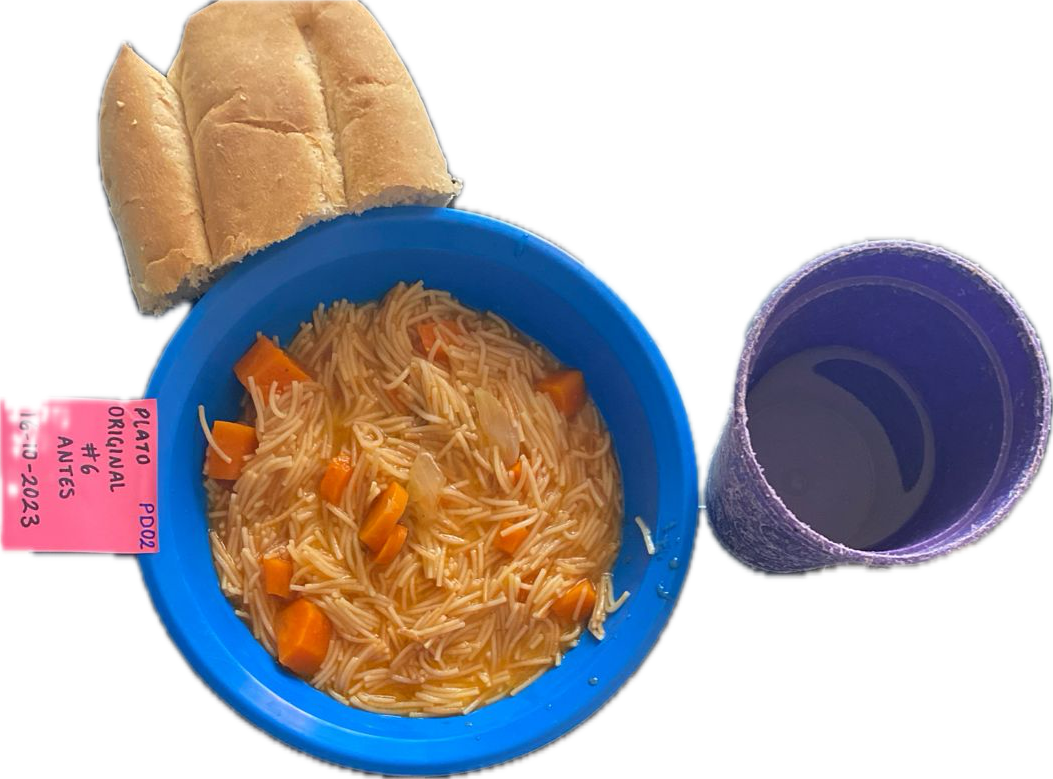 | 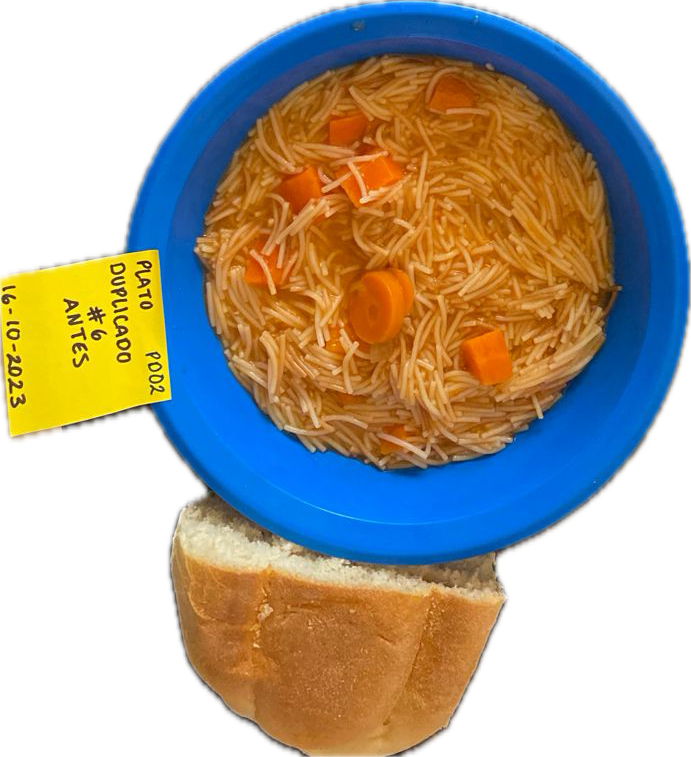 |
